# Novel LIMK2 Inhibitor Blocks Panc-1 Tumor Growth in a mouse xenograft model

**DOI:** 10.18632/oncoscience.7

**Published:** 2014-01-01

**Authors:** Roni Rak, Roni Haklai, Galit Elad-Tzfadia, Haim J. Wolfson, Shmuel Carmeli, Yoel Kloog

**Affiliations:** ^1^ Department of Neurobiology, The George S. Wise Faculty of Life Sciences, Tel-Aviv University, Tel Aviv, Israel; ^2^ The Blavatnik School of Computer Science, Raymond and Beverly Sackler Faculty of Exact Sciences; ^3^ School of Chemistry, Raymond and Beverly Sackler Faculty of Exact Sciences

**Keywords:** LIMK, cofilin, pancreatic cancer, Rho, T56-LIMKi

## Abstract

LIM kinases (LIMKs) are important cell cytoskeleton regulators that play a prominent role in cancer manifestation and neuronal diseases. The LIMK family consists of two homologues, LIMK1 and LIMK2, which differ from one another in expression profile, intercellular localization, and function. The main substrate of LIMK is cofilin, a member of the actin-depolymerizing factor (ADF) protein family. When phosphorylated by LIMK, cofilin is inactive. LIMKs play a contributory role in several neurodevelopmental disorders and in cancer growth and metastasis. We recently reported the development and validation of a novel LIMK inhibitor, referred to here as T56-LIMKi, using a combination of computational methods and classical biochemistry techniques. Here we report that T56-LIMKi inhibits LIMK2 with high specificity, and shows little or no cross-reactivity with LIMK1. We found that T56-LIMKi decreases phosphorylated cofilin (p-cofilin) levels and thus inhibits growth of several cancerous cell lines, including those of pancreatic cancer, glioma and schwannoma. Because the most promising *in-vitro* effect of T56-LIMKi was observed in the pancreatic cancer cell line Panc-1, we tested the inhibitor on a nude mouse Panc-1 xenograft model. T56-LIMKi reduced tumor size and p-cofilin levels in the Panc-1 tumors, leading us to propose T56-LIMKi as a candidate drug for cancer therapy.

## INTRODUCTION

### LIM kinases (LIMK) and cofilin

LIM kinase 1 and 2 (LIMK1/2) are key regulators of the actin cytoskeleton. They phosphorylate and thus inactivate cofilin, a member of the actin depolymerizing factor (ADF) family [1-3]. LIMK1 and 2 are the only two kinases in the LIM protein family [4], which consists of approximately 40 eukaryotic conserved members, all containing the LIM domain named after its initial discovery in the proteins Lin11, Isl-1 and Mec-3. LIMK1/2 each contain two 2 N'-terminal LIM domains responsible for protein-protein interaction [5], a PDZ domain responsible for protein localization, and a C'-terminal kinase domain. They are dual-specificity kinases, phosphorylating both serine/threonine and tyrosine residues [6, 7]. Both are ubiquitous and are expressed in various tissues [8, 9]. They share 50% homology in amino-acid sequence, with higher conservation in specific domains (e.g. 70% sequence homology in the kinase domain). Their expression patterns, however, are different: LIMK1 is expressed mainly in embryonic brain tissue and heart [9], whereas LIMK2 is expressed in all tissues [8]. They also have different subcellular localizations: whereas LIMK1 is mainly localized to the focal adhesion site, LIMK2 is found throughout the cytoplasm and in association with the cis-Golgi compartment [7, 10].

Activation of LIMK1/2 is regulated mainly by the Rho GTPase family of proteins: LIMK2 is activated by the Rho GTPase pathway and is specifically phosphorylated by ROCK (Rho-associated coiled-coil-forming protein kinase) [11, 12], whereas LIMK1 is activated by the Rac-1 GTPase pathway and specifically phosphorylated by PAK1 [13, 14]. PAK1 and ROCK phosphorylate LIMK1 and 2 on threonine residues (T508 and T505, respectively) [11, 15]

The main substrate of LIMK1/2 is the actin-depolymerizing factor (ADF)/cofilin family of proteins, mainly cofilin 1. Although mammals have several cofilin isoforms, cofilin 1 (referred to here as cofilin) is the most abundant isoform in most of the cultured mammalian cell lines [16] and in tumor cell lines [17, 18]. ADF is expressed at much lower levels (5%).

Cofilin plays a prominent role in promoting actin depolymerization [19, 20]. Active (unphosphorylated) cofilin induces severing of actin filaments and participates in numerous cellular functions such as cell migration, cell-cycle processes, and neuronal differentiation [18, 21]. In its phosphorylated state cofilin is inactive and does not affect the cell cytoskeleton. Hyperphosphorylation of cofilin typically occurs in many human diseases and pathological conditions, such as cancer cell invasion and metastases [22], as well as in neurodevelopmental disorders [23], for example Williams syndrome and neurofibromatosis [20, 24-26].

### LIM kinases in cancer and other diseases

LIMKs are major players in cell-cycle progression, cytoskeleton organization, cell migration, and development [10, 27, 28]. Abnormal expression of LIMK1 and LIMK2 is implicated in various malignancies, and is known to be important in tumor manifestation and malignancy. Thus, for example, prostate, breast, and melanoma cancer cells present LIMK1 overexpression [29, 30], whereas LIMK2 was found to be overexpressed in several gene arrays testing changes in gene expression in several cancer types such as ovarian carcinoma, head and neck cancer, and several pancreatic cancer cell lines (CanSER database), and is a predictive marker of drug resistance [31].

Involvement of LIMK1/2 in cancer has become a major focus of interest in the last few years. Recent studies demonstrate an important role for both LIMK1 and LIMK2 in pancreatic tumor progression, cancer cell-induced angiogenesis, and metastasis formation [27]. Vlecken et al. demonstrated that inhibition of LIMK1 and 2 by siRNA inhibits pancreatic cancer metastases in a zebrafish tumor xenograft metastasis assay. The reductions in metastases and in cancer cell-induced angiogenesis were similar for each of the LIMK1 and the LIMK2 single knockdowns. Their double knockdown, however, did not result in complete inhibition and its inhibitory effect was only slightly more pronounced than those of the single knockdowns [27].

LIMK2 is emerging as a cancer cell survival factor that plays a role in chemotherapy resistance in Neuroblastoma cell lines[28, 32] and p53-mediated survival of cancer cells following DNA damage [33]. In addition, the recent finding that LIMK2 is directly activated by Aurora-A, and promoting a positive feedback loop of Aurora-A regulation, suggests that LIMK2 is a key oncogenic effector of Aurora A and that LIMK2 inhibition might be an effective way to inhibit Aurora-A-mediated oncogenic pathways [34].

It was also found that inhibition of LIMK2 in the trabecular meshwork, by promoting depolymerization of the actin filaments, resulted in relaxation of the tissues with consequent increase in outflow facility. The overall outcome was a reduction of ocular hypertension. A similar phenomenon was observed in ROCK knockout mice [35].

LIMKs have also been associated with neurodevelopmental conditions such as Williams syndrome and neurofibromatosis. Chromosome analyses correlate the Williams-Beuren syndrome with a deletion at the LIMK 1 locus[23, 24]. The NF1 protein shown to be a negative regulator of both LIMK1 and 2 [20, 25, 26, 36].

In summary, given their activities described above LIMKs have become persuasive targets for drug design [4, 37, 38]. Their inhibition increases active unphosphorylated cofilin, inhibits cancer metastasis and development, and shows promise as a possible therapeutic measure for LIMK-induced diseases.

### LIMK inhibitors

Despite the importance of LIM kinases in cancer regulation and manifestation, only a few LIMK inhibitors are known. The first to be discovered is BMS-5 [38]. Both LIMK1 and LIMK2 are inhibited by BMS-5 [38]. In 2009, Lexicon Pharmaceuticals discovered a new LIMK2 inhibitor [35]. The most selective compounds were tested *in vivo* in a mouse model of ocular hypertension induced by dexamethasone administration. A single administration of this compound at nanomolar concentration (100 nM) reduced intraocular pressure values to normal. More recently a new LIMK2 inhibitor, Pyr-1, was discovered by cell-based screening that probed the status of microtubule polymerization [39]. Pyr1 was found to be toxic for cancerous cell lines *in vitro* and to inhibit xenografted tumor growth *in vivo*.

Thus, only three cell-permeable inhibitors of LIMK have been identified to date, and only limited experimental data and information on clinical results are available. Development of new inhibitors as potential anti-tumor therapies, as well as for other conditions resulting from overexpression or activation of the Rho-ROCK-LIMK2 pathway, is still a highly desirable objective [7, 35, 38, 40].

We recently reported the development of a new LIMK inhibitor, achieved via computational analysis of the protein structure [36]. We showed that this inhibitor, referred to here as T56-LIMKi, inhibits cofilin phosphorylation, cell growth and migration, and colony formation of NF1-depleted mouse embryonic fibroblasts (MEFs) in soft agar. It was not known, however, whether the T56-LIMKi inhibited LIMK1, LIMK2, or both. That information is important because, as pointed out above, the two LIM kinases have different expression patterns in different tissues or diseases [8, 9]. Here we report that T56-LIMKi is a highly specific inhibitor of LIMK2, and demonstrate its potency in inhibiting growth of pancreatic tumors *in vitro* and *in vivo*.

## RESULTS

### T56-LIMKi inhibits cofilin phosphorylation by LIMK2 but not by LIMK1

**Figure 1:**
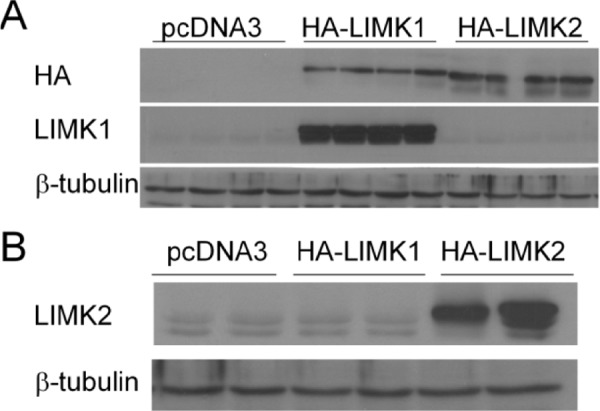
HeLa cells were stably transfected with the PC-vector or with HA-LIMK1 or HA-LIMK2 expression vector (A) Representative blots of the transfected cells obtained with anti-HA or anti-LIMK1 antibodies. (B) Results of the corresponding experiment using anti-LIMK2 antibodies.

In our previous report using the NF1-depleted MEFs we were unable to tell whether T56-LIMKi inhibits LIMK1 or LIMK2 or both. To obtain this information, in the present work we performed *in-vitro* experiments with HeLa cells stably expressing the vehicle (control), LIMK1, or LIMK2. These cells were chosen because they express low levels of p-cofilin [41] and undergo transfection with high efficiency. We transfected HeLa cells with pcDNA3 vectors containing HA-tagged LIMK1 or LIMK2, or with an empty vector (Fig 1). We found that the cells transfected with HA-LIMK1 or HA-LIMK2 indeed expressed the corresponding enzymes (Fig. 1 A and B). The empty vector control exhibited only low basal levels of the LIMK1 and LIMK2 enzymes.

**Figure 2:**
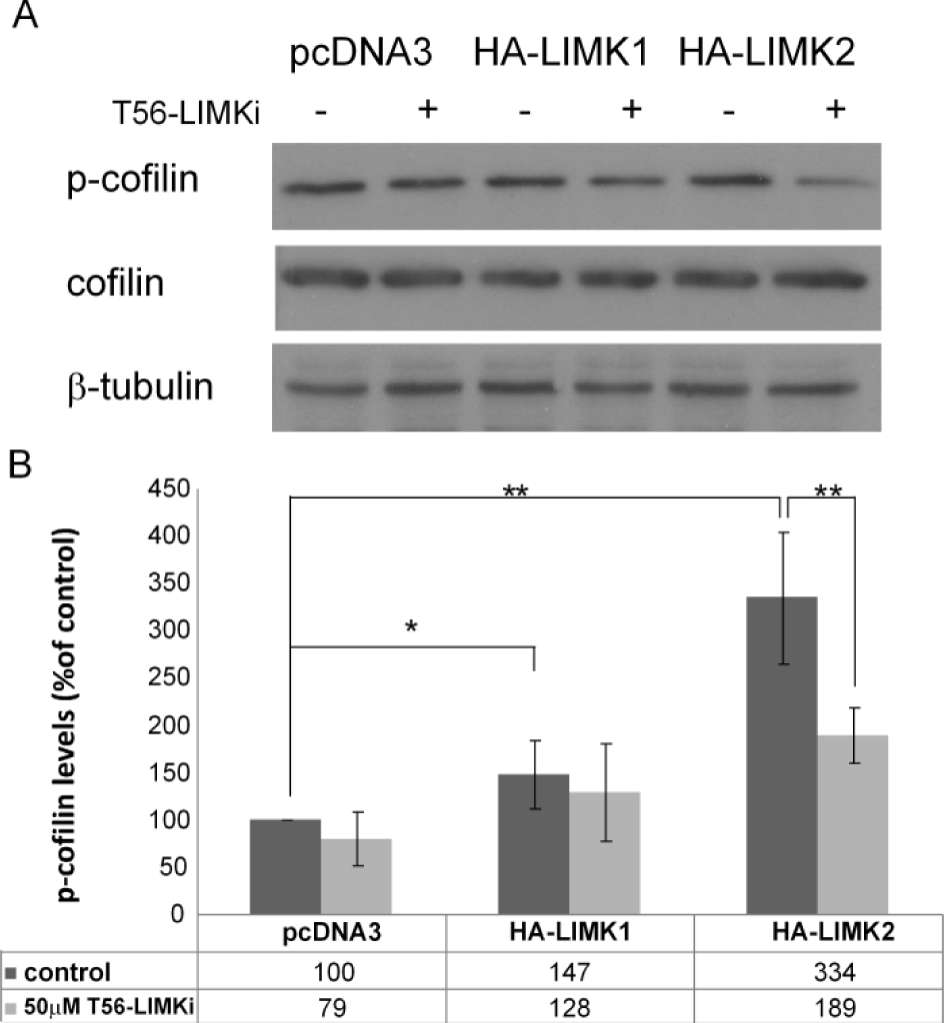
T56-LIMKi inhibits LIMK2-mediated phosphorylation of cofilin HeLa cells stably expressing LIMK1, LIMK2, or vehicle (pcDNA3) were starved for 24 h and then treated with 50 μM T56-LIMKi for 2 h. The resulting p-cofilin, cofilin, and β-tubulin were quantified by Western blotting A. Representative blots. B. Levels of p-cofilin expressed as percentages of untreated pcDNA3 cells, normalized to β-tubulin (means ± SD, *P < 0.05, ** *P* < 0.01).

We then used the stably expressing LIMK1/2 HeLa cells to test T56-LIMKi for specific activity toward one of the LIMKs. Each of the cell lines was treated for 2 h with 50 μm T56-LIMKi or 0.1% DMSO (control). Representative blots and quantifications are shown in Fig. 2. We found that the cells transfected with pcDNA3 exhibited some endogenous p-cofilin which was affected only slightly by T56-LIMKi. Levels of cofilin phosphorylation observed in the LIMK1 transfectants were significantly higher than in the control, and were not inhibited by T56-LIMKi (Fig. 2 *A* and *B* Levels of cofilin phosphorylation observed in the LIMK2 transfectants were also significantly higher than in the control, and this phosphorylation was strongly inhibited by T56-LIMKi (Fig. 2 A and B). Based on these findings, we concluded that the T56-LIMKi is a selective inhibitor of LIMK2.

**Figure 3:**
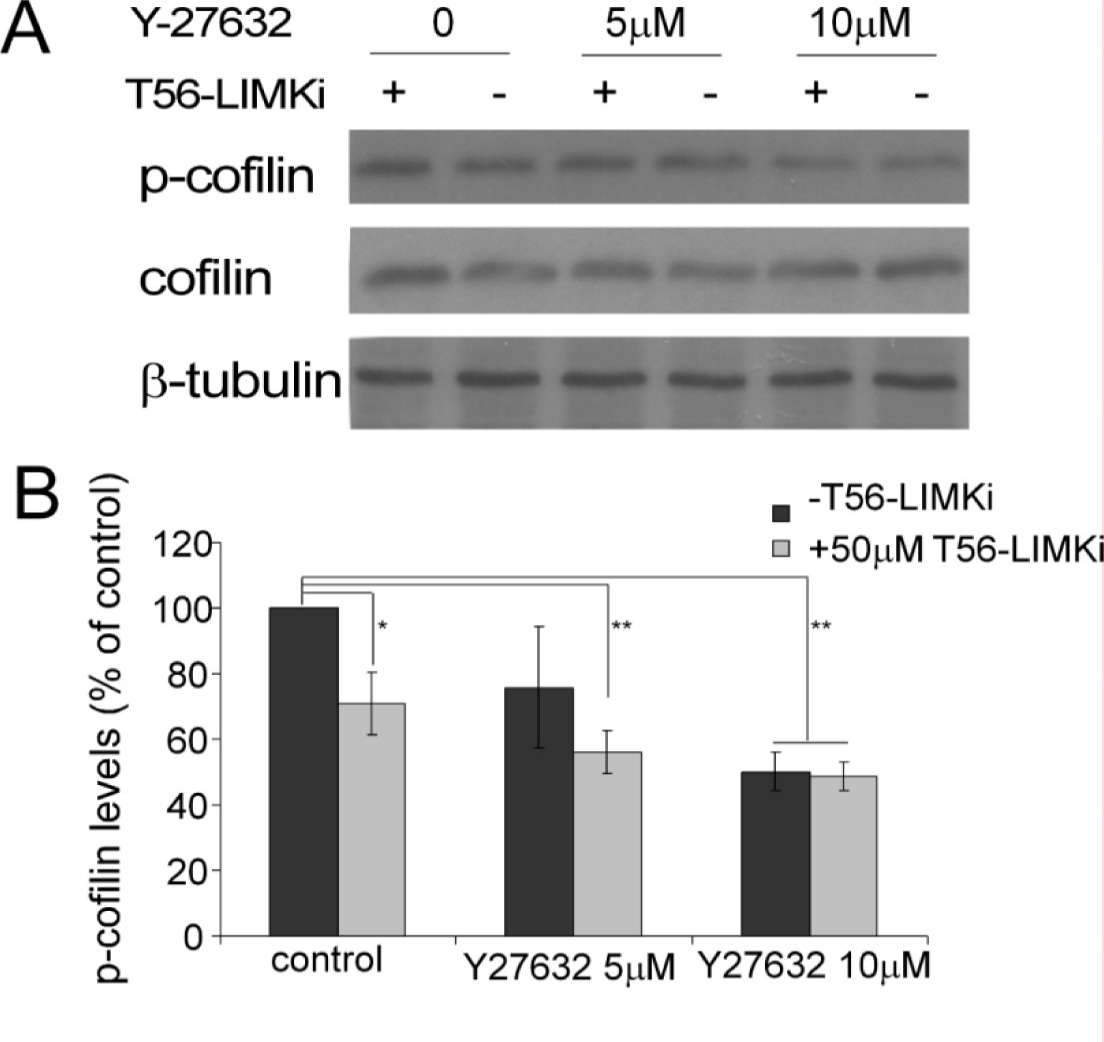
ROCK inhibitor Y-27632 mask T56-LIMKi inhibition of cofilin phosphorylation NF1-depleted MEFs were starved and treated for 24 h with the indicated concentrations of Y-27632 or vehicle (control). The next day, T56-LIMKi 50 μm or 0.1% DMSO was added for 2 h in the presence of 10% FCS. Cells were lysed and analyzed by Western blotting for p-cofilin. A, representative blots. B, quantification of p-cofilin as a percentage of untreated control levels, normalized to β-tubulin (means ± SD, *P < 0.05, ** *P* < 0.01).

To further To verify that LIMK2 is the main substrate of T56-LIMKi, we used the ROCK inhibitor to inhibit upstream activation of LIMK2. ROCK is known to specifically phosphorylate LIMK2, and this activation occurs downstream of RhoA. We treated NF1-depleted MEFs with the ROCK inhibitor Y-27632 and then with T56-LIMKi (Fig. 3). Y-27632 inhibited cofilin phosphorylation in a dose-dependent manner (5 μM Y-27632 inhibited phosphorylation by 25% ± 17%, and 10 μm Y-27632 inhibited it by 50% ± 5.5%). Interestingly, the combined effects of the two inhibitors were additive, and reached saturation at the higher Y-27632 concentration, meaning that at this concentration, adding T56-LIMKi to Y-27632 had no additional inhibitory effect: T56-LIMKi alone inhibited cofilin phosphorylation by 29% ± 9%, and in combination with 5 μM or 10 μM Y-27632 by 44% ± 6.5% or 51% ± 4%, respectively. The fact that T56-LIMKi did not augment the inhibition obtained with Y-27632 suggested that T56-LIMKi acts via the same pathway as Y-27632, namely the RhoA-ROCK- LIMK2 signaling pathway described previously [20]. In line with the observed overexpression of LIMK2 in HeLa cells (Fig. 2), our experimental data (Figs. 2 and 3) suggested that T56-LIMKi is indeed a specific inhibitor of LIMK2. Interestingly, in our previous study using a computational molecular modeling technique, T56-LIMKi was selected based on structure homology of LIMK2 active site to EphA3 inhibitor binding site, which was less conserved in the LIMK1 active site [36]. The present results are in complete agreement with the results of that computational study, thus further verifying that T56-LIMKi is a specific inhibitor of LIMK2.

### T56-LIMKi inhibits cell growth of several cancer cell lines

**Figure 4:**
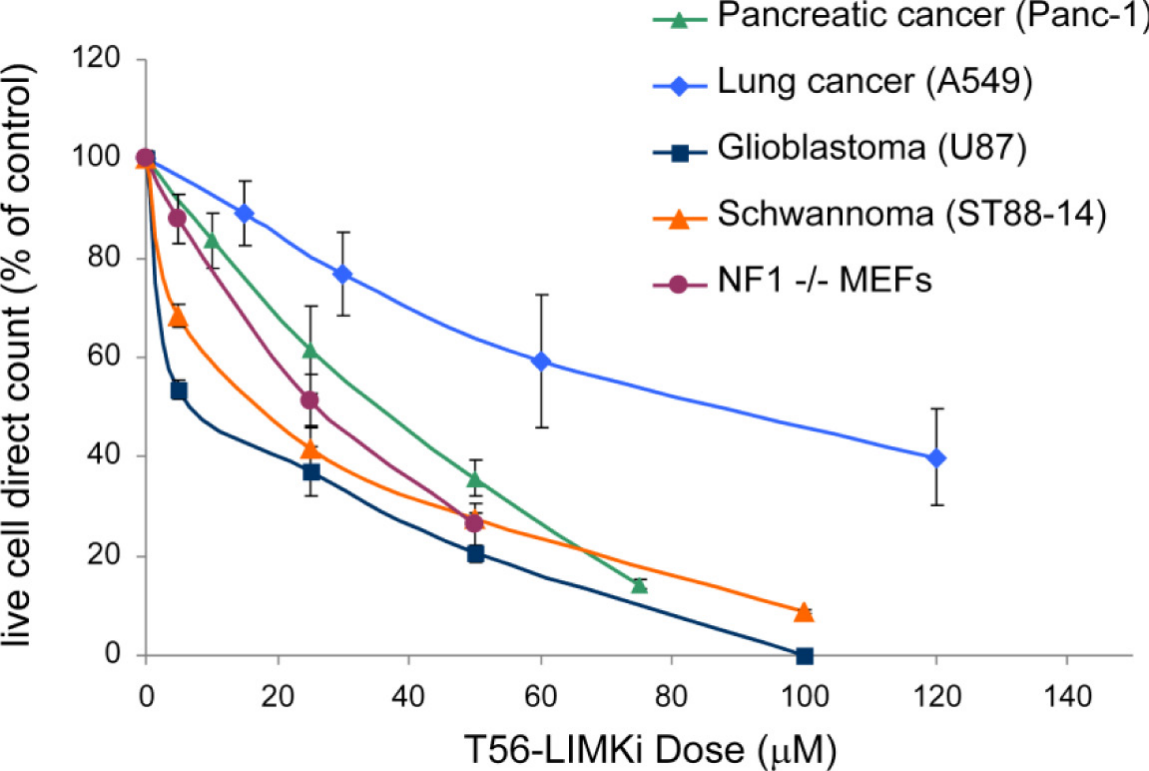
T56-LIMKi inhibits proliferation of various cancer cell lines in a cell-line specific manner Cells were seeded and grown in the absence and in the presence of the indicated concentrations of T56-LIMKi or with 0.1% DMSO (control). After 6 days the cells were then directly counted. Typical inhibition curves are shown (means ± SEM, *n* = 9, *P < 0.05, ** *P* < 0.01).

That LIMK2 plays a prominent role in cancer is a fairly recent finding. LIMK2 is now thought to be an important player in tumor cell invasion and metastasis, especially in pancreatic, breast, and neuronal cancers. We selected a few cancer cell lines for the purpose of measuring in vitro cell growth inhibition by T56-LIMKi. The glioblastoma U87 and the schwannoma ST88-14 cell lines were chosen because their NF1 levels are low, meaning that in those cell lines LIMK2 activity is not downregulated by NF1 [26]. More specifically pancreatic cancer (Panc-1) was selected because LIMK2 was shown to be involved in tumor progression in this cell line [27]. The lung cancer cell line A549 was also selected, because lung cancers are the most common cancer in terms of mortality and incidents and are frequently chosen cancers for drug design [42]. The cells were grown in different concentrations of T56-LIMKi. After 6 days we compared the growth-inhibitory effect of T56-LIMKi on each cell line by direct counting of the cells, and found that T56-LIMKi had efficiently inhibited the growth of ST88-14, U87, and Panc-1 cells with IC50 values of 18.3 ± 5 μM, 7.4 ± 7 μM, and 35.2 ± 5μM, respectively. These values are in the same range as the IC50 values for NF1-/- MEFs. Growth inhibition of the A549 lung cancer cells, however, showed the remarkably high IC50 of 90 ± 14 μM (Fig. 4).

### Inhibition of cofilin phosphorylation in different cancer cell lines

**Figure 5:**
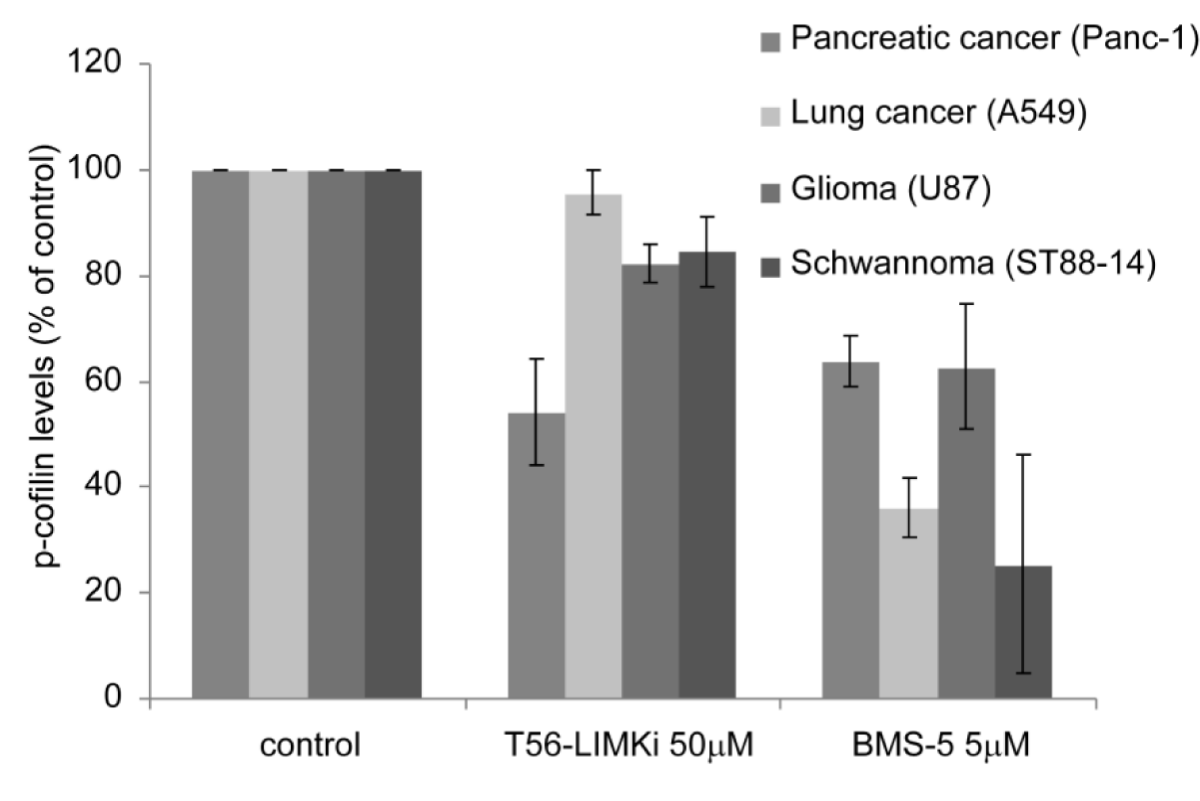
Levels of p-cofilin levels are reduced in a cell-specific manner manner by T56-LIMKi Panc-1, A549, U87, and ST88-14 cells were plated in 10-cm dishes, 5 × 105 cells per plate. The cells were serum-starved for 24 h and then treated with 50 μM T56-LIMKi, 5 μM BMS-5, or vehicle(0.1% DMSO) for 2 h at the indicated concentrations. The cells were then homogenized and their proteins were immunoblotted with specific antibody, quantified, and normalized to p-cofilin, as described in Material and Methods. Average inhibition was calculated from three sets of independent experiments. Results for each cell line are presented as a percentage of the untreated control (mean ± SD, n = 3) All changes observed between controls and treatments, except for T56-LIMKi treatment of A549 cells, were significant (P < 0.01, Student's t-test).

We further tested the ability of T56-LIMKi to inhibit phosphorylation of cofilin in the various cell lines by comparing its inhibitory effect to that of BMS-5, known to inhibit both LIMK1 and LIMK2. The most remarkable T56-LIMKi-induced decrease in p-cofilin among all the cell lines tested was found in the pancreatic cancer cell line Panc-1 (46% ±10%; Fig. 5). Cofilin phosphorylation in Panc-1 was also inhibited by BMS-5 (37% ± 5%); however, unlike in the case of BMS-5 treatment of NF1^−^depleted MEFs [36], BMS-5 was not more effective in reducing p-cofilin levels in Panc-1 cells, although it inhibited both LIMKs. This finding is consistent with the results of a recent study in which double knockout of LIMK1 and LIMK2 had no additional effect on tumor growth or metastasis of the Panc-1 cell line in a zebrafish xenograft model [27]. In a similar manner, U87 cell growth inhibition was relatively high with both treatments, namely, T56-LIMKi and BMS-5 decreased p-cofilin by 24% ± 10% and 38% ± 12%, respectively, with no significant difference between the two treatments. In contrast A549 and ST88-14 cells reacted differently to the two inhibitors: inhibition of their p-cofilin levels by BMS-5 treatment was significantly higher than that observed with T56-LIMKi (75% ± 20% and 65% ± 55, in ST88-14 and A549, respectively). T56-LIMKi decreased p-cofilin only by 20% ± 8% in ST88-14 cells, but hardly decreased it at all (4% ± 4%) in A549 cells (Fig. 5).

In general, our results showed a positive correlation between the cell growth inhibition and the inhibition of cofilin phosphorylation induced by T56-LIMKi. This was indicated by the fact that the T56-LIMKi-induced inhibition of cofilin phosphorylation was lowest in the A549 cells, the cell line that was also the least sensitive in terms of T56-LIMKi-induced growth inhibition.

### Oral T56-LIMKi treatment inhibits human pancreatic tumor growth in nude mice

Our result showed that the pancreatic cancer cell line, Panc-1, had the most remarkable decrease of cofilin phosphorylation among the tested cancers. This finding was in line with previous research on LIMK2 importance in Pancreatic cancers[27, 43]. Therefore, to test the effect of our novel LIMK2 inhibitor on cell transformation *in vivo*, the model we chose was a mouse xenograft injected with Panc-1 cells (a similar experimental paradigm to the one we used to test Salirasib, another inhibitor of human pancreatic tumor growth [44]). As before, the drug was administered orally in 0.5% carboxymethyl cellulose (CMC) gavage, as described in Material and Methods. This technique was selected because of the poor solubility of the compound, which limits its use in aqueous topical formulations or via intraperitoneal injection. T56-LIMKi toxicity in the mice was evaluated after a single administration of 20, 40, 60, 80 or 100 mg/kg. In an initial toxicity study, mice were followed for 2 weeks to monitor possible toxic effects. They showed no weight loss (data not shown) and none of the mice died, indicating that T56-LIMKi is not toxic in nude mice.

**Figure 6:**
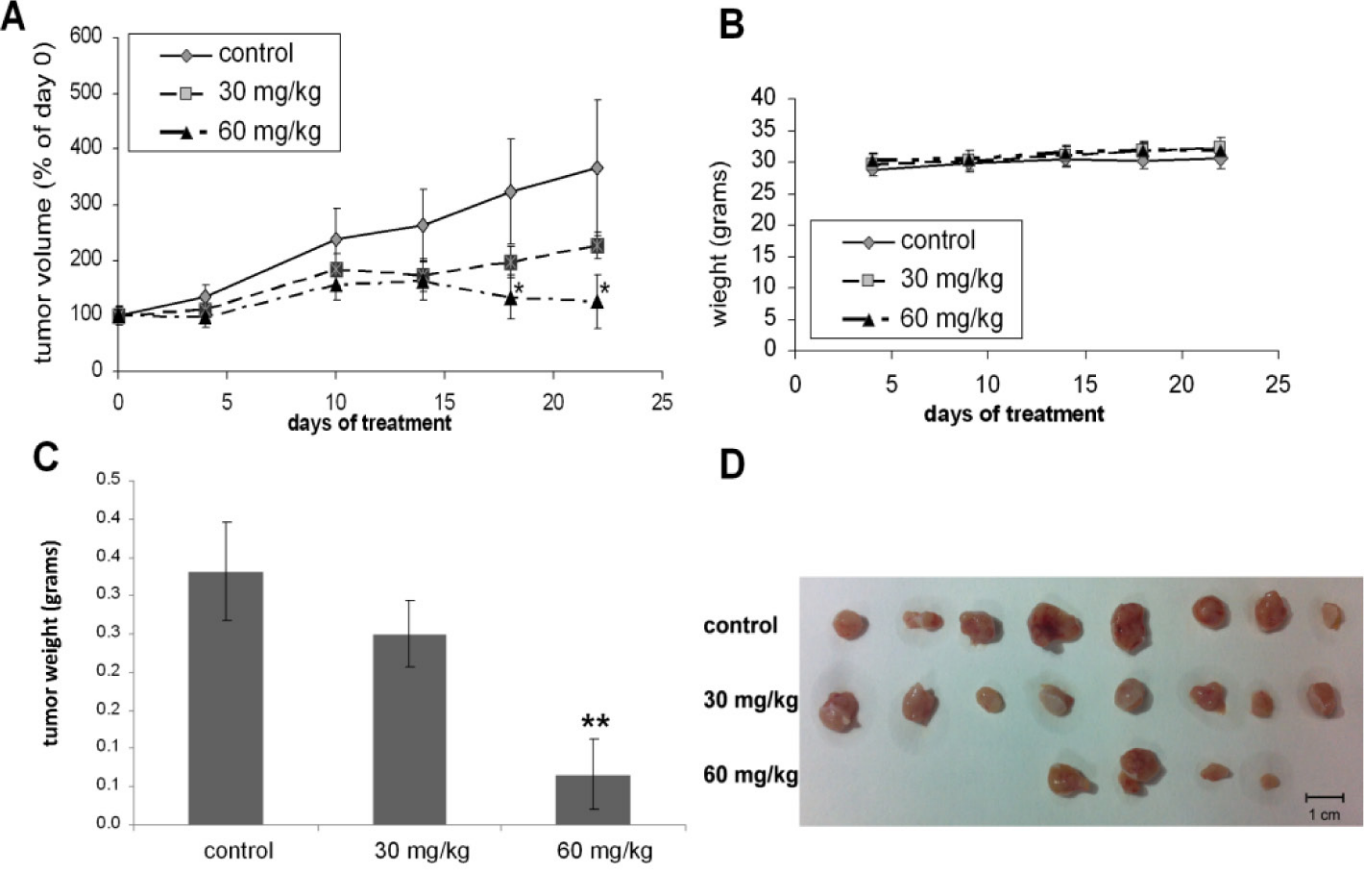
T-56-LIMKi inhibits proliferation of Panc-1 tumor cells in nude mice A, Panc-1 cells were implanted s.c. in the right flank of nude mice and the mice were divided into treatment groups and treated with T-56-LIMKi as described in Materials and Methods. Tumor volumes (means ± S.E.M, * P <0.05, n=8) during the treatment period are shown. B, Mice were weighed at the indicated times during the experiment. C and D, after 35 days of treatment tumors were removed, photographed, and weighed. (means ± S.E.M).

Following the toxicity study, mice were implanted with 5×10^6^ xenografted Panc-1 cells subcutaneously (s.c.) in the right flank. Treatment was started 7 days later (day 0 of the treatment), when the mice were separated randomly into three groups. Mice in the two experimental groups (*n* = 8 per group) were each treated with a daily oral non-toxic dose of T56-LIMKi (30 or 60 mg/kg in gavage) and mice in the control group (*n* = 8) received only the vehicle (0.5% CMC) in the gavage. The results of this experiment showed a dose- and time-dependent decrease in tumor volume (Fig. 6A). Mice treated with T56-LIMKi (60 mg/kg) showed a significant decrease in tumor volume compared to control (Fig. 6B). After treatment for 35 days tumors were removed, weighed, and homogenized for immunoblot assays. Tumor size relative to the control group was found to be significantly decreased. Of the eight tumors in the group treated with T56-LIMKi at 60 mg/kg, four disappeared completely, two were reduced in size by about 80%, and the remaining two were not affected at all (Fig 6C, D). This experiment was performed twice with similar results.

**Figure 7:**
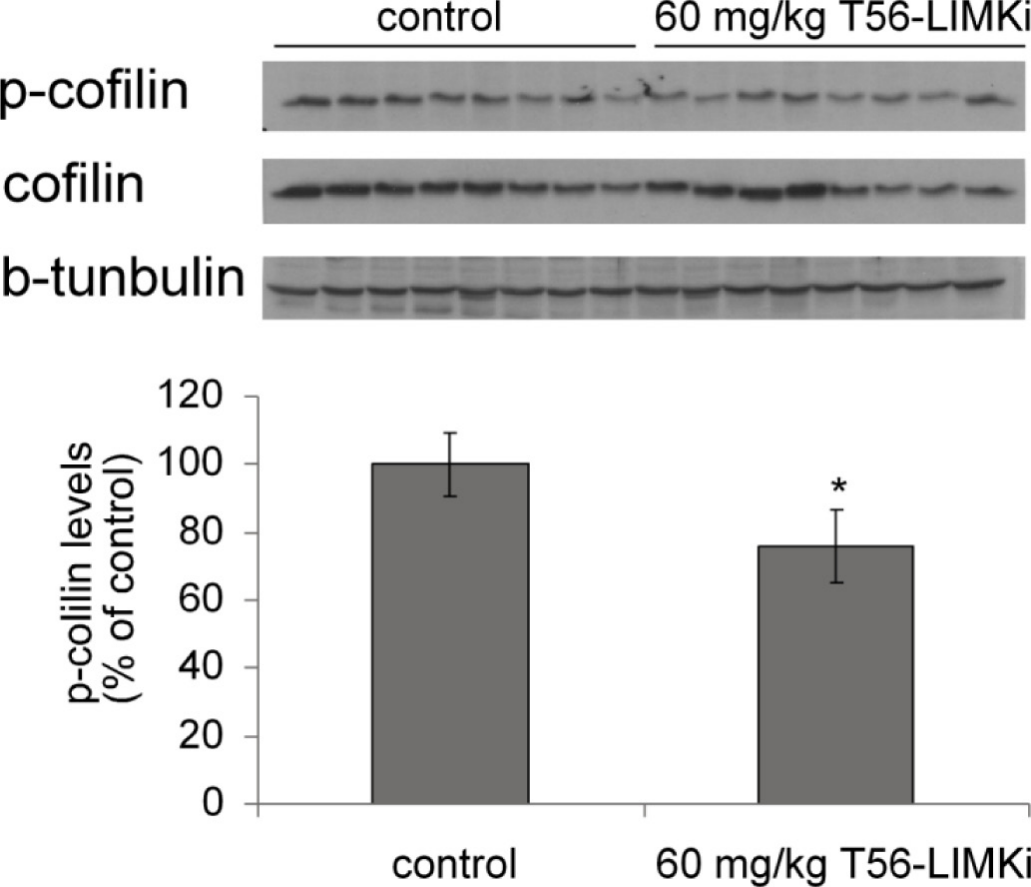
T56-LIMKi reduces p-cofilin levels in Panc1- cell xenografts in a nude mouse model Levels of p-cofilin were determined in lysates from eight tumors of mice treated with T56-LIMKi (60 mg/kg) and in tumors of vehicle-treated control mice. A, Immunoblots; B, quantification of the p-cofilin leves normalized to total cofilin (means ± S.E.M, n=8, * P <0.05).

Next we determined the levels of p-cofilin in homogenates from the T56-LIMKi-treated and control tumors. We found that in homogenates prepared from mice treated with T56-LIMKi at 60 mg/kg, p-cofilin levels 35 days after the start of treatment were reduced relative to control by 25 ± 10.8% (*n* = 8, *P* < 0.05, Student's t-test; Fig. 7). We conclude that T56-LIMKi can induce inhibition of cofilin phosphorylation and Panc-1 tumor shrinkage *in vivo*.

## DISCUSSION

We recently reported the development of a new LIMK inhibitor, T56-LIMKi, that inhibited cofilin phosphorylation, cell growth and migration, and colony formation of NF1-depleted MEFs in soft agar [36]. It was not known, however, whether the T56-LIMKi inhibited LIMK1, LIMK2, or both.

To gain a better understanding of the inhibitory activity of T56-LIMKi on cofilin phosphorylation, we first examined whether T56-LIMKi is a general inhibitor of both the LIMK1 and the LIMK2 actin polymerization regulators or inhibits only one of them. This knowledge is essential for understanding the mechanism of action of T56-LIMKi, and for improving our ability to predict other malignancies and diseases that might treatable by this novel inhibitor. In this study we first addressed this question by overexpressing LIMK1 or LIMK2 in HeLa cells, in which p-cofilin levels are relatively low [41]. We found that T56-LIMKi inhibited cofilin phosphorylation in cells that overexpress LIMK2 but not LIMK1 (Fig. 1). In line with these results, we found that inhibition of ROCK using the known ROCK inhibitor Y-27632 acted additively and not synergistically with T56-LIMKi, and that their combined effect reached a plateau corresponding to about 50% inhibition of cofilin phosphorylation (Fig. 3). This showed that both of these inhibitors act via the same pathway, namely, RhoA-ROCK-LIMK2. Based on these experiments, we concluded that T56-LIMKi is a selective inhibitor of LIMK2.

Since LIMK2 is known to play a prominent role in cancer, we wanted to find out whether T56-LIMKi inhibits tumor growth and tumor cell death. To address this question we chose a number of diverse cancer cell lines, namely pancreatic cancer, lung cancer, glioma and schwannoma. LIMK2 reportedly plays a role in tumor manifestation in pancreatic cancer, glioma and schwannoma cells [26]. A549 cells were selected as a LIMK2 non-related cancer cell type, but based on the Lung cancer prevalence Worldwide [42].

We found here that T56-LIMKi inhibited phosphorylation of cofilin in Panc-1, U87, and ST88-14 cells (Fig. 5). It did not, however, inhibit cofilin phosphorylation of A549 cells, as expected because in these cells LIMK2 is not over-activated. Accordingly, we found that T56-LIMKi strongly inhibited cell growth of all three of the above mentioned cell lines (IC50 < 30 μM) but did not strongly affect A549 cell growth (IC50 > 90 μM) (Fig. 4). Despite the good correlation between T56-LIMKi inhibition of cofilin phosphorylation and cell-growth inhibition, we cannot rule out the possibility that T56-LIMKi inhibition of cell growth might also result from an effect of T56-LIMKi on other targets, such as EphA3, a receptor homologues to LIMK2 [36].

Our examination of pancreatic cancer (Panc-1) tumors xenografts in mice indicated that T56-LIMKi was indeed able to prevent tumor growth at doses that are well tolerated by the tested mice (Fig. 6). Panc-1 cells presented the highest sensitivity to T56-LIMKi inhibition of cofilin phosphorylation and a relatively low IC50 (Fig. 4 and Fig. 5). T56-LIMKi inhibited their tumor formation *in vivo* in a dose-dependent manner and reduced p-cofilin levels of the tumor cells with no sign of attendant toxicity (Fig. 6 and Fig. 7).

Based on all of the above results, we suggest that T56-LIMKi has promising therapeutic potential for acting as a safe inhibitor of cancer cells characterized by LIMK2 over-activation or by over-activation of LIMK2 upstream signaling pathways such as RhoA [20], P53 [33], and Aurora-A kinase pathways [34]. We propose that T56-LIMKi is a potential drug for pancreatic cancer, glioma and schwannoma cells.

## MATERIALS AND METHODS

### Materials

Compound T5601640 (defined here as T56-LIMKi) was purchased from Ambinter (Paris, France). The LIMK inhibitor BMS-5 (Bristol-Myers Squibb) was purchased from SYNkinase (Shanghai, China). ROCK inhibitor Y-27632 was purchased from R&D Systems (Minneapolis, MN). The antibodies used were rabbit anti cofilin , rabbit anti phospo-cofilin (Ser3) and rabbit anti LIMK1 (Cell Signaling Technology, Beverly, MA); rabbit anti-β-tubulin antibody (Sigma-Aldrich, St. Louis, MO); mouse anti-HA antibody (Covance, Berkeley, CA); rabbit anti-LIMK2 (Abcam, Cambridge, MA ); peroxidase-conjugated goat anti-rabbit and goat anti-mouse IgG (Jackson ImmunoResearch, West Grove, PA).

### Cell culture

NF1 knockout mouse embryonic fibroblasts (MEFs) were prepared from NF1+/- mice, as described previously [45]. MEFs, HeLa, Panc-1, and U-87 cells were grown in Dulbecco's modified Eagle medium (DMEM) containing 10% fetal calf serum (FCS), 2 mM L-glutamine, 100 units/mL penicillin, and 100 μg/ml streptomycin. Cells were incubated at 37°C in a humidified atmosphere of 95% air and 5% CO2. The human NF1 MPNST cell line ST88-14 was obtained from Dr. Nancy Ratner (University of Cincinnati) and maintained in RPMI/15% FCS. A549 cells were maintained in Nutrient Mixture F-12 Ham containing 10% FCS, 2 mM L-glutamine, 100 U/ml penicillin and 100μg/ml streptomycin. For biochemical and Western blotting assays, cells were plated at 5×10^5^ cells per 10-cm dish or at 15×10^4^ cells per 6-well plate, and for immumofluorescence at 7500 cells per 18-mm glass coverslip. For growth inhibition assays, cells were plated at 5×10^3^ per 24-well plate. All treatments are specified in the text or figure legends.

### Western blot analysis

Cells were plated at a density of 5 × 10^5^ cells in 10-cm dishes and were allowed to grow overnight in medium containing 10% FCS. After 24 h the medium was replaced with medium containing 0.5% FCS, and the cells were treated for 2 h with T56-LIMKi at the indicated doses. The cells were then lysed with solubilization buffer (50 mM Tris-HCl pH 7.6, 20 mM MgCl_2_, 200 mM NaCl, 0.5% NP40, 1 mM dithiothreitol, and protease inhibitors), and the lysates (50 μg) were subjected to SDS–PAGE and then immunoblotted with one of the following antibodies: anti-p-cofilin) (1:1000), anti-cofilin (1:1000), and anti-β-tubulin (1:500). Immunoblots were then exposed to peroxidase-conjugated goat anti-rabbit IgG (1:2500), and protein bands were visualized by enhanced chemiluminescence and quantified by densitometry (EZ-Qant).

### Transfections

To establish stably transfected Hela clones, cells were transfected using Jetpei (Polyplus-transfection SA, Illkirch, France) according to the manufacturer's protocol. Cells were transfected with plasmid containing either LIMK1 or LIMK2 fused to HA-tagged appropriate empty vectors, all with G418 resistance. At 48 h after transfection the medium was replaced with with growth medium containing 1 mg/ml G418. A polyclonal batch was collected and validated by Western blotting. All stable cell lines were maintained in DMEM containing 10% FCS and 400 μg/ml G418 and were grown for not more than 2 months.

### Construction of plasmids and vectors

HA-tagged pcDNA3-LIMK1 and HA-tagged pcDNA3-LIMK2 were a kind gift from Prof. Hélène Bénédetti, Centre National de la Recherche Scientifique, Orleans, France.

### Dose preparation and animals

A solution of 0.5% carboxymethylcellulose (CMC) solution was prepared as follows. Double- distilled water (approx. 60 ml) was added to 0.5% w/v CMC. After mixing for 4 h with a magnetic stirrer the solution was transferred to a volumetric flask and the volume was adjusted to 100 ml. The solution was kept at 4 °C. T56-LIMKi was stable in aqueous CMC for at least a week. Before drug administration the suspension was warmed to room temperature with continuous stirring.

Nude CD1-Nu mice (6 weeks old) were housed in barrier facilities on a 12-h light/dark cycle. Food and water were supplied ad libitum. On day zero, 5 × 10^6^ Panc-1 cells in 0.1 ml of PBS were implanted Subcutaneous (s.c.), just above the right femoral joint. S.c. tumors were measured with a caliper, and animal weights were recorded every 4-5 days. Tumor volumes were calculated using the formula: [length × width] × [(length + width)/2]. When tumor volumes reached 0.06−0.07 cm^3^ (day 0 of treatment), the mice were randomly separated into three groups. Control mice were fed with CMC (vehicle), and T56-LIMKi-treated mice received either 30 or 60 mg/kg of T56-LIMKi daily (by oral administration of 0.1 ml with 0.5% w/v CMC) For imunoblot procedures, tumors were weighed and then homogenized (10% w/v) in lysis buffer as detailed previously [46]. Total amounts of p-cofilin, cofilin and β-tubulin were determined in samples (60 μg protein) of each lysate by SDS-PAGE followed by immunoblotting with the specific antibodies All animal procedures were in accordance with International Laws and Policies.

### Statistical calculations

Statistical significance was calculated using one-tailed Student's *t* test.

### Image processing

Images were adjusted for brightness/contrast and were cropped by Adobe Photoshop.
